# RBP-Maps enables robust generation of splicing regulatory maps

**DOI:** 10.1261/rna.069237.118

**Published:** 2019-02

**Authors:** Brian A. Yee, Gabriel A. Pratt, Brenton R. Graveley, Eric L. Van Nostrand, Gene W. Yeo

**Affiliations:** 1 Department of Cellular and Molecular Medicine, University of California at San Diego, La Jolla, California 92093, USA; 2 Institute for Genomic Medicine, University of California at San Diego, La Jolla, California 92093, USA; 3 Bioinformatics and Systems Biology Graduate Program, University of California at San Diego, La Jolla, California 92093, USA; 4 Department of Genetics and Genome Sciences, Institute for Systems Genomics, UConn Health, Farmington, Connecticut 06030, USA

**Keywords:** alternative splicing, eCLIP, splicing map, RNA binding protein

## Abstract

Alternative splicing of pre-messenger RNA transcripts enables the generation of multiple protein isoforms from the same gene locus, providing a major source of protein diversity in mammalian genomes. RNA binding proteins (RBPs) bind to RNA to control splice site choice and define which exons are included in the resulting mature RNA transcript. However, depending on where the RBPs bind relative to splice sites, they can activate or repress splice site usage. To explore this position-specific regulation, in vivo binding sites identified by methods such as cross-linking and immunoprecipitation (CLIP) are integrated with alternative splicing events identified by RNA-seq or microarray. Merging these data sets enables the generation of a “splicing map,” where CLIP signal relative to a merged meta-exon provides a simple summary of the position-specific effect of binding on splicing regulation. Here, we provide RBP-Maps, a software tool to simplify generation of these maps and enable researchers to rapidly query regulatory patterns of an RBP of interest. Further, we discuss various alternative approaches to generate such splicing maps, focusing on how decisions in construction (such as the use of peak versus read density, or whole-reads versus only single-nucleotide candidate crosslink positions) can affect the interpretation of these maps using example eCLIP data from the 150 RBPs profiled by the ENCODE consortium.

## INTRODUCTION

After RNA is transcribed from DNA, intronic regions are removed and exons are joined together in the process of splicing. Most exons are constitutively spliced, meaning they are always included in the mature RNA transcript that is ultimately translated. However, recent estimates indicate that nine out of every 10 human genes undergo alternative splicing in which alternative splice sites are utilized in a cell type- or condition-specific manner to create distinct RNA transcripts from the same pre-mRNA molecule (Wang et al. 2008). The key role of alternative splicing is further confirmed by the linkage of splicing regulation to numerous human diseases, including neurological disorders and many types of cancer (Scotti and Swanson 2016). Thus, understanding the regulatory patterns that control alternative splicing can give valuable insights into a variety of biological systems.

RNA binding proteins (RBPs) interact with RNA through recognition of sequence motifs, structures, and combinations thereof to regulate condition-specific alternative splicing. Thus, identifying the direct in vivo targets of RBPs can give insight into their mechanism of regulation. Most commonly, this is done through cross-linking and immunoprecipitation (CLIP), which pulls down an RBP of interest along with its bound RNA (Lee and Ule 2018). However, although in vivo targets in isolation can yield insights into potential roles for an RBP, integration of this data with RBP-responsive targets allows the identification of directly regulated targets, which can provide a deeper understanding of the mechanisms of regulation by an RBP. For regulation of alternative splicing, where RBP binding can cause either inclusion or exclusion of alternative exons, it is common to identify RBP-responsive events by knocking down or over-expressing the RBP and performing RNA-seq. Following sequencing, several algorithms have been developed to discover changes in splicing among transcripts between conditions (Katz et al. 2010; Shen et al. 2014). These algorithms detect common splicing events, including skipped exons (SE), alternative 3′ and 5′ splice sites (A3SS, A5SS), retained introns (RI), and mutually exclusive exons (MXE), all of which contribute to increased diversity of the human proteome.

In addition to simple overlaps between the lists of RBP-responsive events and RBP-bound regions, it has become common to specifically query how the positional dependence of binding differentially affects alternative splicing of nearby events. Visualization of this location-dependent splicing regulatory information is often referred to as a “splicing map,” which has become an important tool to visualize RNA binding activity over a collective set of genomic regions (Witten and Ule 2011). For a given splice type, a meta-event is typically shown to visualize global binding across alternatively spliced (AS) exons containing a composite signal across a set of events. These meta-events are often comprised of vectorized windows aligned to the splice site and the flanking proximal exon/intron region (Yeo et al. 2009; Lim et al. 2011; Hauer et al. 2015). For example, SE events are typically represented as four windows showing all upstream splice sites, all 5′ and 3′ splice sites of the cassette exon, and downstream splice sites, plus corresponding surrounding regions (usually 50 nt into the exon, 200–300 nt into the intron) (Xue et al. 2009; Witten and Ule 2011; Cereda et al. 2014; Park et al. 2016). Analogous approaches can be used to visualize the other types of splicing events as well, focusing on the splice site regions associated with the meta-event type.

In particular, splicing maps which overlap RBP binding with AS events from RNA-seq provide insight into how RBPs can regulate these events differently depending on where they associate (Witten and Ule 2011). Early on, these maps were made by using motif enrichment as a proxy for RBP association, showing for example that the RBFOX family of RBPs appeared to encourage exon inclusion if associated downstream but increased exon exclusion if associated upstream (Yeo et al. 2009). However, these approaches are limited to RBPs with well-characterized binding motifs, which remains a small subset of RBPs overall. The use of CLIP to profile protein–RNA interactions directly has led to a rapid expansion of this area, with maps generated for a variety of RBPs that reveal a complex regulatory specificity for RBPs based on their location of binding (Witten and Ule 2011). However, due to the variety of CLIP-seq technologies and their current limitations, there remains a lack of consensus on numerous details regarding the calculations underlying the generation of such maps (Wheeler et al. 2017; Lee and Ule 2018).

Recently, the ENCODE project published eCLIP data sets for 150 RBPs across K562 and HepG2 cell types, as well as identification of alternative splicing for shRNA knockdown of 263 RBPs in HepG2 and K562 cell types (Van Nostrand et al. 2016, 2017a). As part of this effort to perform integrated analyses of RNA processing to map splicing regulatory patterns, we observed that many decisions could significantly alter the interpretation of the subsequent splicing map. Here, we describe RBP-Maps (https://github.com/yeolab/rbp-maps) a robust software tool to standardize the generation of these maps from ENCODE and other data sets in order to enable integration of CLIP and RNA-seq for nonexpert users. Further, we discuss numerous analysis options enabled by this tool, and how these decisions can shape the downstream generated splicing map.

## RESULTS

### Generation of splicing regulatory maps with RBP-Maps

**FIGURE 1.**
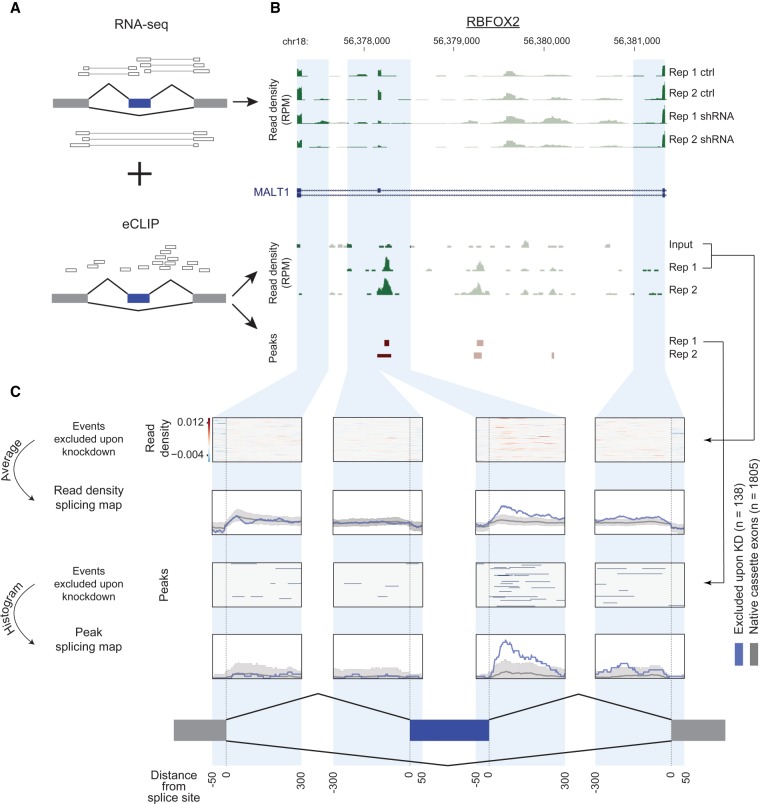
Splicing maps reveal position-dependent correlation between RBP binding and RBP-responsive targets. (*A*) Models showing (*top*) RNA-seq junction reads quantitating exon inclusion or exclusion and (*bottom*) eCLIP reads identifying “peaks” as regions of enrichment. (*B*) Example derivation of a splicing map. (*Top*) RNA-seq read density (in reads per million [RPM]) in RBFOX2 shRNA knockdown and (*bottom*) RBFOX2 eCLIP read density and peaks (enriched in immunoprecipitation versus paired input) for exon 7 in MALT1 (ENST00000348428.3) in HepG2 cells. (*C*) Integration across 138 RBP-responsive (excluded upon knockdown) events yields an averaged splicing map for (*top*) read density or (*bottom*) peak density.

To enable simplified generation of splicing regulatory maps for the ENCODE eCLIP and RNA-seq data sets, we developed the RBP-Maps software package. At its core, the program intersects a CLIP data set provided by the user (either read densities in the form of bigwig coverage files or peaks in the form of bed files) and intersects them with any number of user-defined alternative splicing event files (Fig. 1A). The program then outputs a normalized summary figure displaying the average signal across all events (Fig. 1B), including data matrices containing the raw and normalized signal values for each event, as well as the mean across all events, for each position in the meta-exon map as a comma separated file in order to facilitate further downstream processing. The RBP-Maps package is publicly available (https://github.com/yeolab/rbp-maps), and contains details regarding installation setup requirements, usage, and examples for different alternative event types (including cassette or SE, A5SS, A3SS, MXE, and RI).

As an essential component of this software, we have also provided a number of additional options, including read density normalization, window size, density outlier removal options, statistical significance calculation, and incorporation of multiple background event lists. In the following sections, we discuss how each of these options can affect the resulting splicing map and provide recommendations for usage.

### Avoidance of duplication within RBP-responsive events

The first decision in the generation of a splicing map is the selection of set(s) of alternative events, which is specified by the “--annotations” option. By default, alternative splicing input files are in the rMATS JunctionCountsOnly.txt file format (for SE, RI, A3SS, A5SS, and MXE event types). However, support is also available for the MISO format (Katz et al. 2010; Shen et al. 2014), and alternative events from other splicing quantitation tools can be used by simply reformatting the list into the rMATS event output format. Any number of event lists can be provided, each of which will be separately processed and plotted together in the subsequent splicing map. This enables the user to include not only the experimental event set (for example, events included or excluded upon RBP knockdown), but also various control sets of constitutive or alternative exons not responsive to the RBP for comparison purposes (see further discussion below).

**FIGURE 2.**
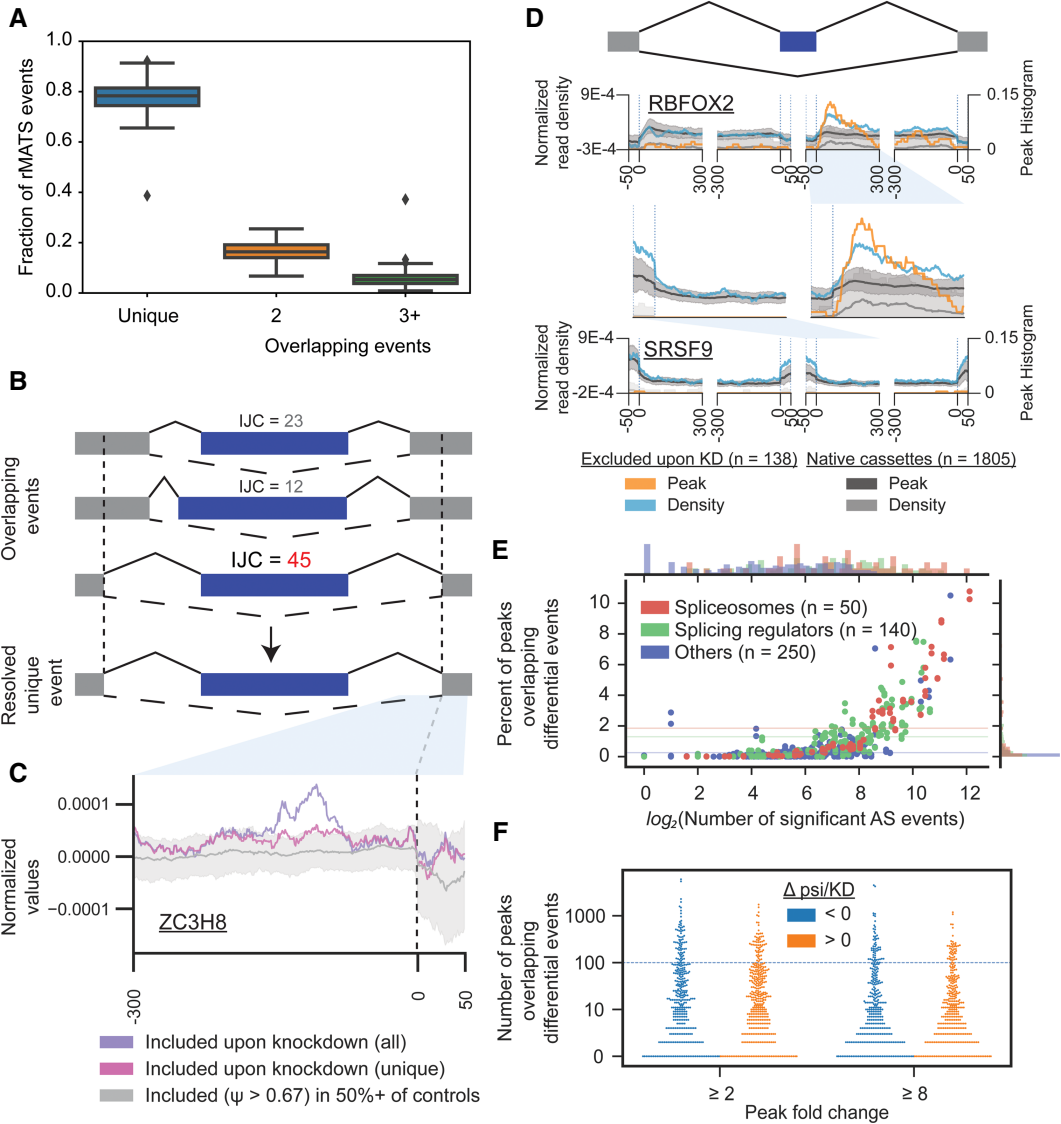
Event-driven options in creating splicing maps. (*A*) Boxplot indicates the distribution across 473 RBP knockdown RNA-seq data sets separated into included and excluded sets of events for the fraction of event regions that overlap one, two, or three or more differential event calls identified by rMATS. (*B*) Schematic indicates multiple overlapping events within one event region. The event with the highest IJC is kept as the resolved “unique event.” (*C*) Example splicing map for ZC3H8 (in K562 cells) showing the difference in the resulting map made by either (purple) including all rMATS-identified differential events or (pink) after discarding overlapping events. A set of natively included cassette exons which show exons that are included (≥67% percent spliced in/Ψ) in at least 50% of control RNA-seq experiments is shown in gray. (*D*) Splicing maps shown for (*top*) RBFOX2 in HepG2 and (*bottom*) SRSF9 in HepG2 cells. Maps made based on density of significantly enriched eCLIP peaks are shown in orange, with maps made using read density shown in blue. (*E*) Points indicate (*x*-axis) the number of significant RBP-responsive AS events versus (*y*-axis) the fraction with eCLIP peaks overlapping the event. Colors indicate RBP function annotations. (*F*) Violin plot indicates the number of RBP peaks overlapping RBP-responsive events for 203 eCLIP and knockdown RNA-seq comparisons. Shown are distributions for peaks at least twofold or eightfold enriched in IP versus paired input.

We have found that outputs from many standard RNA-seq splicing analysis tools require pruning of events in order to avoid duplication of CLIP signals in the resulting splicing map, as some software reports multiple “events” for the same gene that in fact overlaps (often due to these events sharing one [or multiple] exon–exon junctions). As a consequence, distinct splicing events can share genomic coordinates, which would result in integrating the same eCLIP signal multiple times in the subsequent regulatory map. We observed that these overlapping events accounted for an average of 22% of the total number of events reported by rMATS among all submitted ENCODE data sets (Fig. 2A), suggesting that there could be significant double- (or more) counting of single eCLIP peaks if overlapping events were not removed. Therefore, we group overlapping events and select the event with the highest inclusion junction count (IJC) as the resolved unique event to be incorporated into the splicing map using the included subset_rMATS_ junctioncountsonly.py script (Fig. 2B). We observed that the most common source of these events were exons which shared exclusion junctions and had variable 5′ or 3′ splice sites, many of which had extremely low inclusion levels.

To show the effect on an example splicing map, we considered ZC3H8 in K562 cells. Plotting Z3CH8 CLIP against an unfiltered set of 239 events identified as significantly included (change in percent spliced in (|ΔΨ|) ≥ 0.05, FDR ≤ 0.1 and *P* ≤ 0.05) upon knockdown of ZC3H8 causes a peak in the global signal in the proximal upstream intron of the meta-downstream exon (Fig. 2C). However, this appears to be the result of intersecting CLIP signal multiple times across 50 overlapping events, as this peak was no longer observed when we removed overlapping events. Thus, we have found that such double-counting of eCLIP signal can cause artifacts in splicing maps if not properly accounted for.

### Reads versus peaks

There are two major alternative approaches to how CLIP signal is utilized in a splicing map: as either read density (in various processed or normalized forms) or as the density of significantly enriched peaks. To enable researchers to implement both of these approaches, RBP-Maps can run in two modes: --peak mode (which takes a bigbed file that describes significantly enriched regions of CLIP signal identified from any standard CLIP analysis toolkit), and --density mode (which accepts read densities formatted as two standard bigwig files, one for each strand).

Conversion of read density into computationally identified peaks or clusters, using one of a variety of peak-calling algorithms, is a standard step of CLIP analysis (De and Gorospe 2017). The use of peaks provides two appealing benefits for simplified creation of splicing maps. First, because peaks identify regions where immunoprecipitation (IP) signal is significantly enriched over a background model, they mitigate noise in read density signal by focusing on regions of significant enrichment (Park et al. 2016). Second, by compressing the CLIP signal to a single binary value indicating the presence of a peak at each position, each event is weighted equally in the resulting average signal trace, removing the need for further normalization of the CLIP signal to control for relative abundance or differential enrichment.

For peak-based maps, a count of peaks that overlap AS regions is plotted as a histogram at every position, with the final value as the fraction of events that contain a peak at each position (i.e., the total count divided by the number of regions). Considering the ENCODE data set, we observe that a subset of RBPs show clear splicing maps based on peak density alone: For example, RBFOX2 shows enrichment for peaks downstream from the 5′ splice site of knockdown-excluded exons (Fig. 2D). Thus, for some sufficiently deeply sequenced CLIP data sets from proteins with distinct binding patterns, peak-based maps offer the most succinct way of integrating CLIP and alternative splicing data (Fig. 2D). These RBPs typically bind directly to, or near splice sites and provide high position-specific overlap between splice events and CLIP peak regions, yielding a consistent signal across the meta-event.

However, we noted that other RBPs had results that varied between peak- and read-based maps. For example, a map based on read density for SRSF9 in HepG2 cells showed enrichment at knockdown-excluded exons, consistent with the general role of SR proteins as enhancing exon inclusion (Ibrahim et al. 2005). However, a peak-based map provides limited insight, as only four knockdown-excluded exons are overlapped by a significantly enriched reproducible eCLIP peak (Fig. 2D). This is a common occurrence among ENCODE data sets, as the mean percentage of peaks intersecting RBP knockdown-altered SE regions (3′ end of the upstream exon to the 5′ end of the downstream exon) is <1% (with a slightly higher average of 1.3% and 1.9% for known splicing regulators and spliceosomes, respectively), limiting the power of the peak-based approach (Fig. 2E). Even decreasing the stringency threshold for peak identification from eightfold to only twofold enriched in IP versus input background yields only a slight increase in the median number of peaks overlapping events from 4 to 12 (Fig. 2F). Thus, even though peak-based maps are often simpler (both conceptually as well as computationally), read density-based maps remain highly useful due to increased signal (Fig. 2D). In both cases, 50–100 or more AS events are generally required to yield robust maps.

### Read density-based approaches: normalization

Splicing regulatory maps based off of read density are generated by RBP-Maps --density mode, in which the user data are provided as bigwig format read density files (one for each strand) for both IP and (if available) paired input (or other control) experiments. However, read density does not inherently include normalization against background or provide regions of enrichment as compared to peaks. Therefore, we have made three CLIP density normalization options available as part of --density mode in RBP-Maps: “raw” values (option [0]), which illustrates a splicing map using just CLIP read densities (and is the same method used for peak-based maps), “subtraction” normalization (option [1], default for density), which subtracts normalized size-matched input read densities from its corresponding CLIP IP read density, and “entropy” normalization (option [2]), which calculates information content-based fold enrichment of CLIP read density over corresponding input.

**FIGURE 3.**
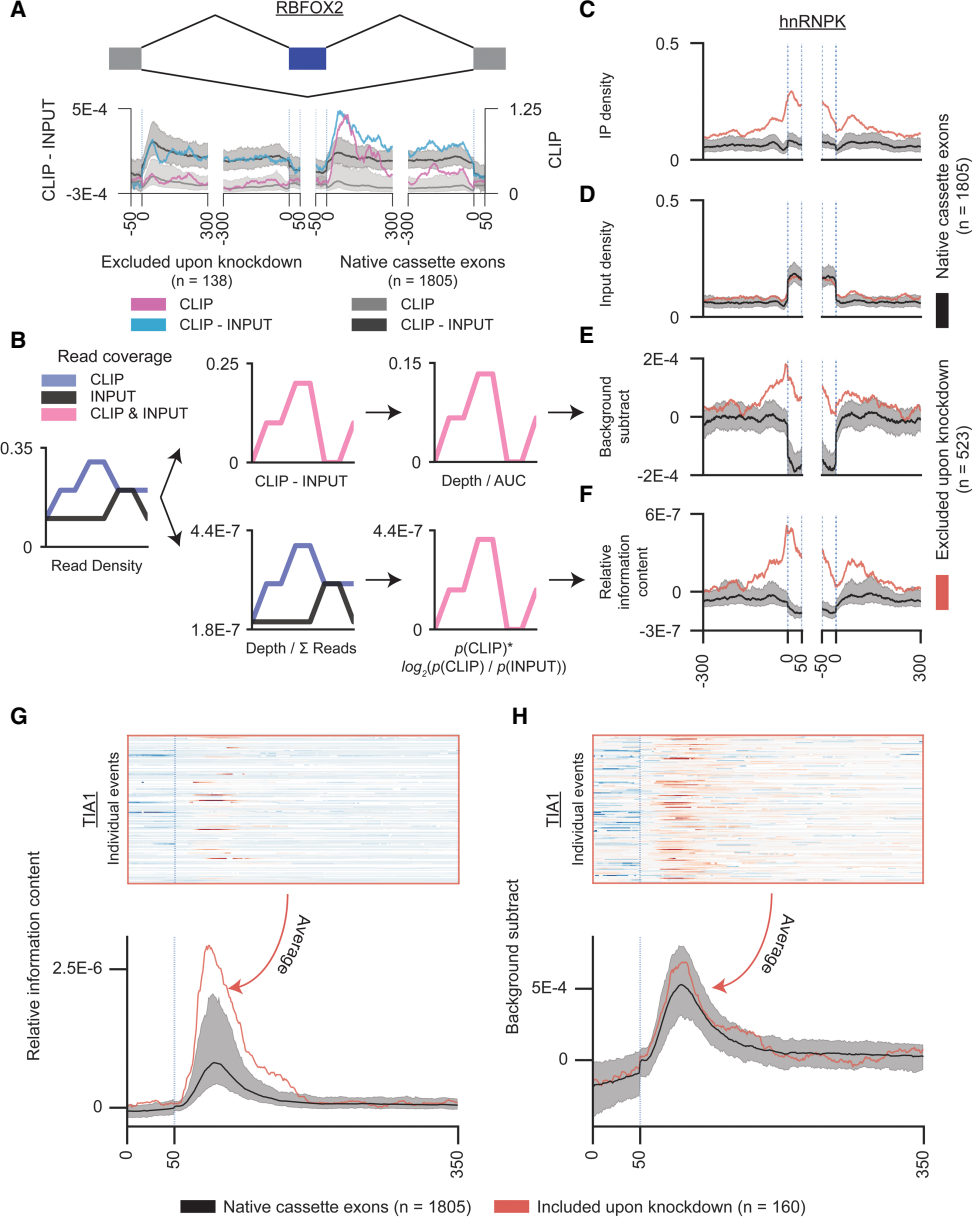
Strategies for normalizing read density maps against an input background. (*A*) Curves indicate splicing maps generated for cassette exons excluded upon RBFOX2 knockdown using either (pink) eCLIP read density alone or (blue) normalized read density after comparing eCLIP read density versus size-matched input sample. (*B*) Schematic of the “background subtraction” versus the “information content” normalization for a single example event. (*Top*) In the “background subtraction” approach, input read density is subtracted from immunoprecipitation (IP) read density, then is normalized against area under the curve represented by read density. (*Bottom*) In the “information content” approach, read density is normalized to the fraction of total reads in the data set, followed by calculation of a relative information value at each position between IP and input. (*C–F*) Lines indicate differences observed upon generating splicing maps for excluded events upon HNRNPK knockdown using different inputs and normalization methods: (*C*) read density in eCLIP only, (*D*) read density of size-matched input only, (*E*) “background subtraction” normalization, and (*F*) “information content” normalization. (*G*) Heatmap shows (*top*) information content-normalized values and (*bottom*) corresponding average across the 5′ splice site region of a meta-cassette exon for TIA1 (HepG2). (*H*) As in *G*, but shown for background-subtraction normalized values.

We observe that in some instances, density maps look similar regardless of normalization method. This is particularly true in cases where size-matched input background is low, as is the case for intronic-binding RBPs that are often being profiled in studies of splicing regulatory networks. For example, subtracting input from the CLIP signal from an RBFOX2 skipped/cassette exon map yields little differences in peak position, aside from changes in scaling (Fig. 3A). However, experiments that include a size-matched input can leverage this information to correct for common background artifacts, including the typical observation of nonenriched read density at abundant exonic regions (Van Nostrand et al. 2016). Indeed, applying different normalization methods to an HNRNPK splicing map does change the shape of binding upstream and downstream from the cassette exon (Fig. 3C–F). Thus, although using IP read density only can yield reasonable splicing regulatory maps, incorporation of a paired input is recommended.

To consider the effect of normalization methods, we compared two strategies: background subtraction and a “relative information” enrichment method based on an adaption of relative entropy (Fig. 3B). The subtraction method first calculates the difference between density values of the IP and its corresponding input, then scales these values for each event to sum to one, equalizing each region's contribution to the overall splicing map in a manner similar to existing normalization methods (Licatalosi et al. 2008). This prioritizes the global relative shape of binding enrichment (Fig. 3E). In contrast, we tried a second method in which we did not normalize per event, but instead calculated the relative information in IP versus input at each position for each event as

$$p_{i}\times \mathrm{lo}g_{2}\left(\frac{\;p_{i}}{q_{i}}\right),$$

based off Kullback–Leibler divergence, where *p*_*i*_ and *q*_*i*_ are the fraction of total reads in IP and input, respectively, which map overlapping position *i*. The final averaged map was then calculated as the position-wise mean over these information scores across all events (Fig. 3F). This relative information approach maintains the strength of binding, meaning that events with greater read density will dominate the final average.

As expected, we found that the summarized map using the information content-based method would often be highly dominated by highly abundant CLIP signals at only a small number of events (Fig. 3G). In contrast, the subtraction method proved to be an effective approach, yielding more robust signals than peak-based maps while being more resistant to over-weighting single events (Fig. 3H). Thus, the subtraction method provides a mechanism to incorporate paired size-matched input into the standard read density-based splicing regulatory map framework. Although we focused on normalization against a paired input here, we note that these approaches can be similarly applied to compare enrichment against paired IgG or knockout controls. However, other experimental designs may require customization as necessary.

### Outlier removal

**FIGURE 4.**
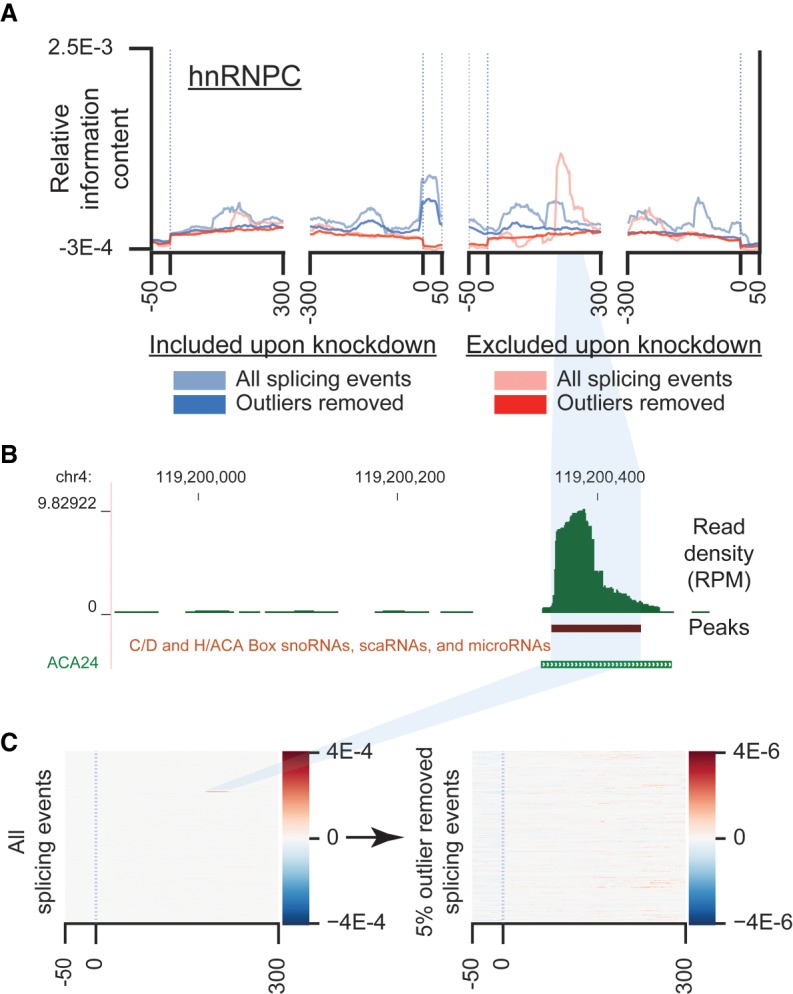
Removing outliers removes local artifacts that may confound global signal. (*A*) Figure shows splicing map of HNRNPC in HepG2 either including all events or excluding outliers (defined as the top and bottom 2.5% of values at each position). (*B*) Genome browser track shows an example outlier, HNRNPC HepG2 eCLIP read density at *ACA24*. (*C*) Heatmaps indicate normalized density tracks for all HNRNPC knockdown-excluded events (*left*) before and (*right*) after removal of outliers.

Particularly with the relative information content method, we observed that individual highly abundant positions at single events could dominate the composite signal. Manual inspection suggested that these typically arose from snRNAs, miRNAs, and other multicopy or highly abundant transcripts or pseudogenes present within these intronic regions. For example, we observed a single site of significant enrichment ∼250 bp downstream from knockdown-excluded SE in HNRNPC splice maps (Fig. 4A). Upon further inspection, we noticed that this signal came exclusively from a single event near a snoRNA (Fig. 4B). To address this, we performed outlier removal on the top and bottom 2.5% signal at each position across each splicing map, which removed extreme outliers and revealed signal consistent with the splicing-repressive role of HNRNP proteins (Fig. 4C). While keeping the middle 95% of values appears to work in removing these artifacts in most ENCODE data sets, the (--conf) parameter can be adjusted to define an alternative outlier threshold. Although this was more critical in generating reliable maps using the relative information metric, we found that it also tended to decrease noise when using the background-subtraction method as well.

### Choice of background events for comparison

Interpretation of a splicing map requires the use of some sort of background control in order to contrast binding of the RBP around RBP-responsive exons to a set of nonresponsive ones. Many studies have indicated that the typical alternative exon is fundamentally different from a typical constitutively spliced exon, with altered exon and intron size, weaker 5′ and 3′ splice sites, higher sequence conservation, and higher RBP binding (Yeo et al. 2005). Thus, although the process of selection of these background events is often treated as so basic as to not warrant further discussion in publications, we have found that the selection of a proper background for comparison is essential for proper interpretation. To explore the effect of comparison against different event backgrounds, we generated five sets of control exons: constitutive exons (which had no exclusion observed in any of 29 scrambled shRNA control RNA-seq data sets in HepG2 or 29 in K562), “native” cassette exons that were AS under normal conditions (0.05 < inclusion < 0.95 in at least half of control RNA-seq data sets), and three subgroups of native events: “included native” (inclusion > 0.67 in at least half of control data sets), “central native” (0.33 < inclusion < 0.67 in at least half of control data sets), and “excluded native” (inclusion < 0.33 in at least half of control data sets).

**FIGURE 5.**
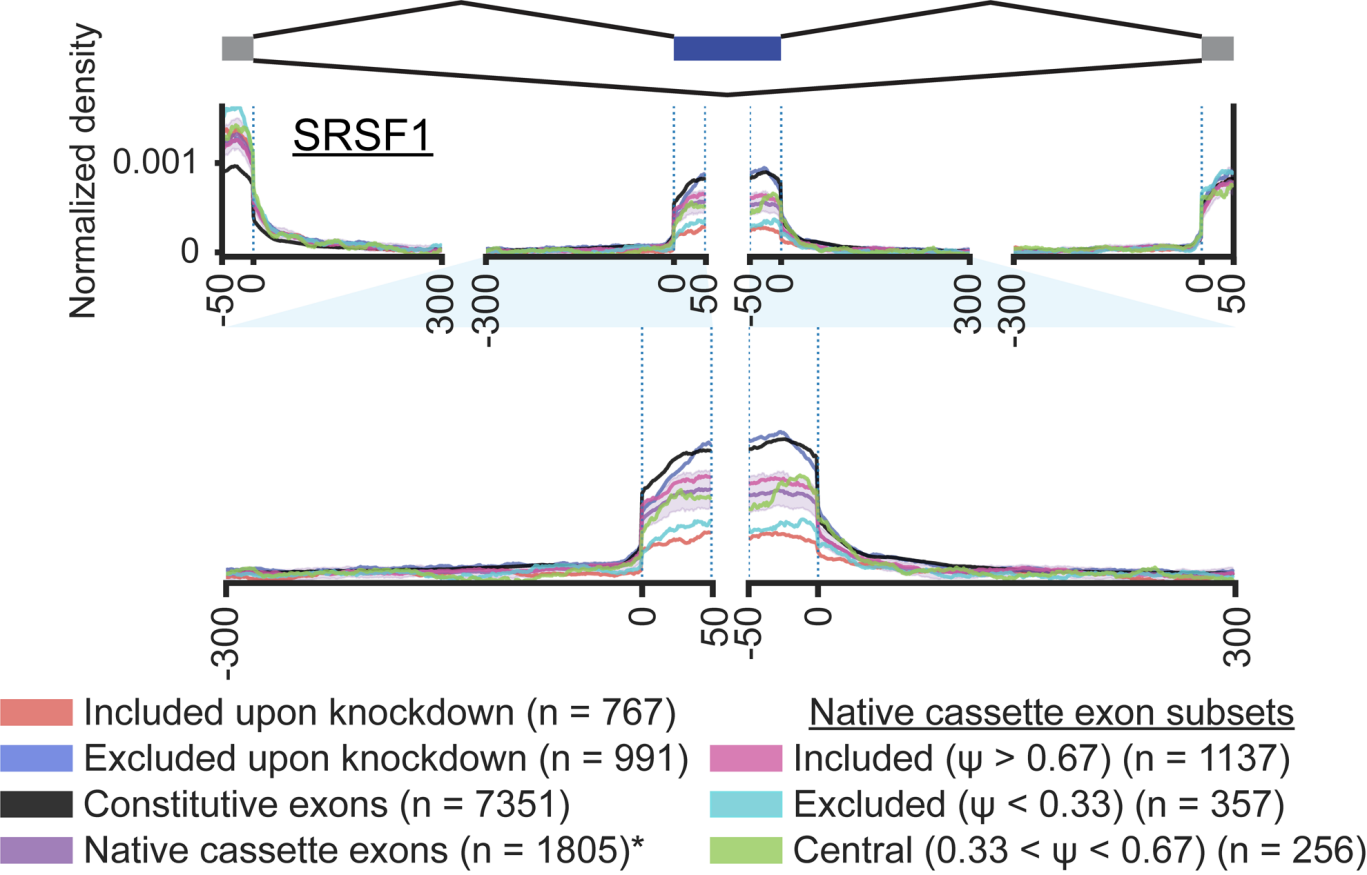
Choice of background affects interpretation of splicing maps. Lines indicate average normalized eCLIP signal at SRSF1 (red) knockdown-included and (blue) knockdown-excluded cassette exon events against four controls: constitutive exons (with no exclusion reads across multiple control RNA-seq data sets), native cassette exons with 0.05 < percent spliced in (Ψ) < 0.95 in at least half of ENCODE control RNA-seq data sets, and subsets of native cassette exons with average Ψ < 0.33 (excluded), 0.33 < Ψ < 0.67 (central), and Ψ > 0.67 (included) in ENCODE control RNA-seq data sets.

As an example of the effect of background choice, we considered the splicing regulatory maps of serine- and arginine-rich splicing factor 1 (SRSF1). We observe that SRSF1 eCLIP signal is higher at exons excluded upon SRSF1 knockdown than those included upon SRSF1 knockdown, consistent with its known role in exon inclusion (Fig. 5; Ibrahim et al. 2005; Zhou and Fu 2013). Next, considering the SRSF1 eCLIP signal at these different background event lists, we observed a clear pattern where exons with higher average inclusion (constitutive or native included groups) had higher SRSF1 eCLIP signal, whereas those with lower inclusion (native excluded) had far less SRSF1 eCLIP signal. Further, we observed that whereas exons excluded upon SRSF1 knockdown had higher SRSF1 eCLIP signal relative to any of the four cassette exon classes, they had equivalent SRSF1 signal to constitutive exons (Fig. 5). This clearly demonstrates the impact of background choice, as if the background selected was largely composed of constitutive exons one might believe that SRSF1 is unchanged at knockdown-excluded events, whereas the use of a native cassette exon background indicates enriched SRSF1 binding at knockdown-excluded events. As the latter conclusion is better supported by the differences between alternative and constitutive exons more broadly as well as previous knowledge about the exon inclusion promoting the role of SRSF1, we believe (based on this and other examples) that using a background of native alternative exons is preferred in nearly all situations.

### Statistical significance models

**FIGURE 6.**
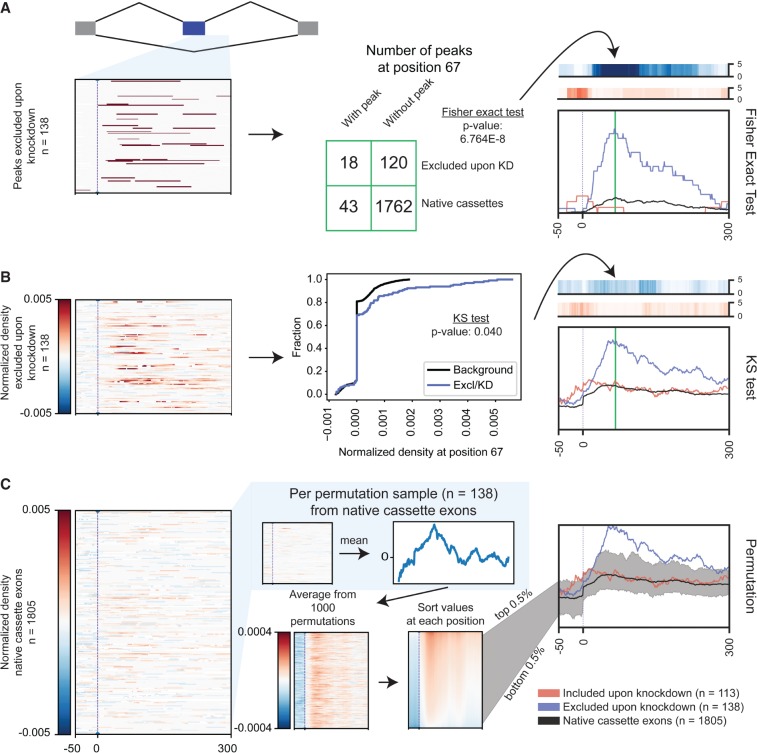
Significance models for splicing maps based on peak versus read density. (*A*) Schematic shows calculation of significance for peak-based splicing maps. (*Left*) Peak positions are mapped across a set of significantly altered events (data shown are for exons excluded upon RBFOX2 knockdown in HepG2 cells). (*Center*) At each position, a Fisher's exact (or equivalent) test is performed between this set and some control set (e.g., native cassette exons; see further discussion in Fig. 5). (*Right*) Resulting significance can be plotted for all positions in the splicing map for (blue) knockdown-excluded or (red) knockdown-included events. Significance is shown on a −log_10_ scale. (*B*) Significance calculation for read density maps using Kolmogorov–Smirnov test. (*Left*) Normalized density is calculated for all knockdown-excluded events. (*Center*) At each position, the distribution of normalized density is compared between knockdown-excluded and a control (native cassette exons). (*Right*) Region-wide results are summarized similar to *A*. (*C*) A bootstrapping strategy identifies confidence intervals for the control event list. (*Left*) Normalized density is identified for the set of native cassette exons. (*Center top*) For each of 1000 permutations, a random sample of events is chosen (matching the number of knockdown-excluded events) and used to generate an average density map. (*Center bottom*) Average maps are collected for all 1000 permutations and sorted at each position to identify 0.5% and 99.5% confidence bounds for the final map. (*Right*) Native cassette exon density maps (along with confidence window) are then plotted along with maps identified from knockdown-excluded and included events.

Once the proper background has been selected, RBP-Maps can test up to two conditions (i.e., significantly included and significantly excluded cassette events) and show position-wise significance against an indicated background using the --sigtest and --bgnum options (which select the 0-indexed number order corresponding to the events to test and the set of events to use as a background, respectively). Different models are used for the peak-based and density-based approaches. For peak-based maps, a Fisher's exact test is used at each position along the meta-event to test whether the fraction of events with a peak at that event is significantly altered relative to the selected background using the “--sigtest fisher” option (Fig. 6A). For density-based maps, users can perform a Kolmogorov–Smirnov test (--sigtest ks), which provides users a way to visualize significance as a heatmap of *P*-values (Fig. 6B). However, we found that this test was not ideal as it tended to yield false positive significance for data sets with many altered events, and conversely was poor at identifying positions (such as the +67 position for RBFOX2) where many events showed no change but a subset showed dramatic change (Fig. 6B). Therefore, we implemented an additional nonparametric test (--sigtest permutation) by performing a random sampling (*n* = 1000) of a chosen background (typically the native cassette exon set). This allows users to generate confidence bounds and significance based on a null distribution of samples of alternative events, which better captures the true variability in signal (Fig. 6C).

### Whole-reads versus 5′ read ends

**FIGURE 7.**
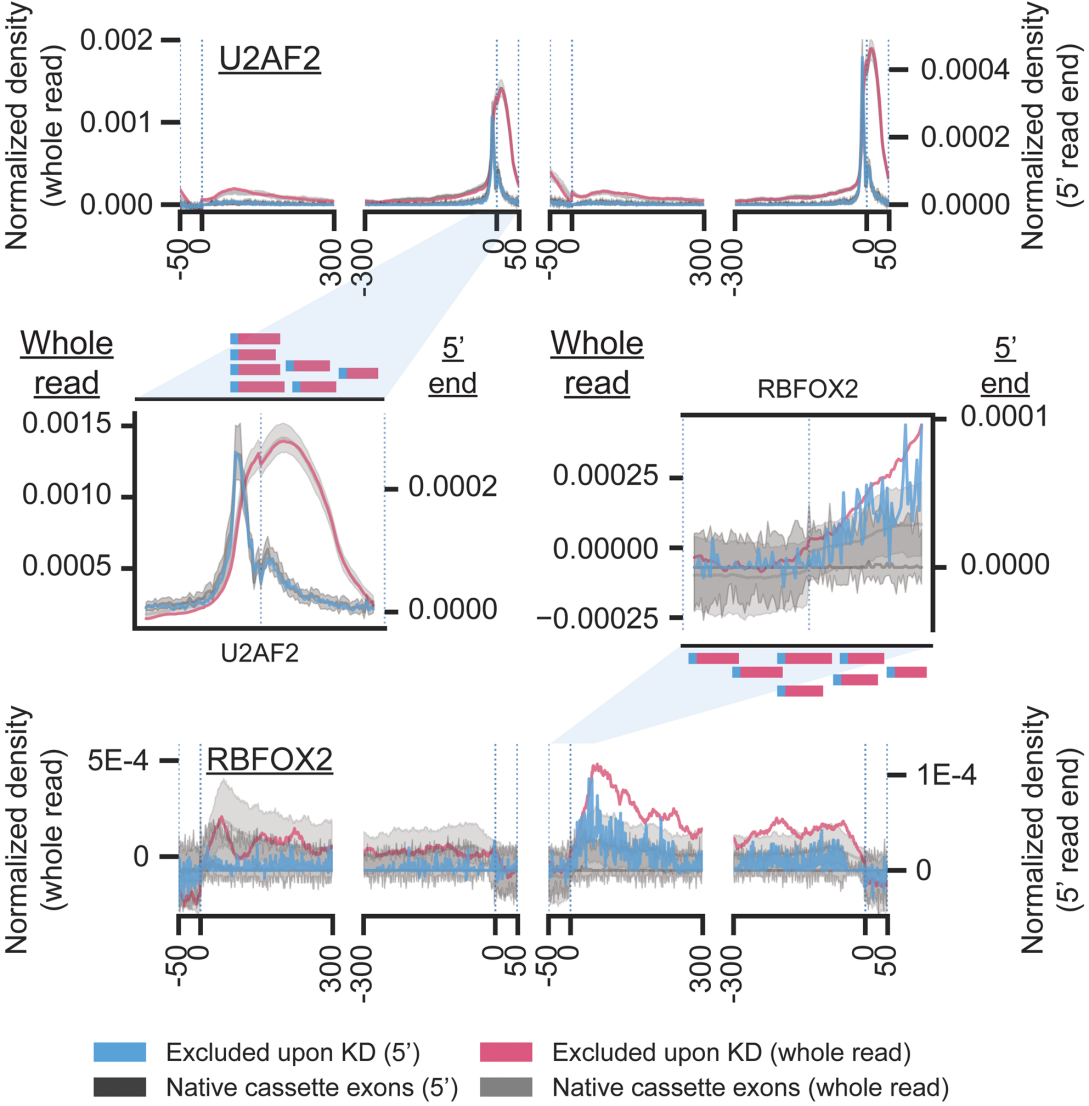
5′ read-based splice maps improve resolution of binding for some RBPs. Shown are splicing maps for (*top*) U2AF2 HepG2 eCLIP signal at exons excluded upon U2AF2 knockdown in HepG2 cells or (*bottom*) RBFOX2 HepG2 eCLIP signal at exons excluded upon RBFOX2 knockdown in HepG2 cells. Splicing maps were generated (red) using the entire read (as in previous figures), or (blue) using the 5′ terminal position of reads only.

During CLIP, reverse transcriptase enzymes often terminate at the site of protein–RNA crosslinking, which causes the 5′ end of reads to correspond to the site of RBP–RNA interaction (with some variability due to the positioning of available crosslinkable amino acids and bases within the binding site) (König et al. 2010; Van Nostrand et al. 2017b). Thus, an additional advantage to the use of read density is the ability to utilize these crosslink-diagnostic events to improve the resolution of the resulting splicing map (König et al. 2010; Wang et al. 2010). To test this, we re-generated splicing maps using just the 5′ ends of each read, and observed variable results depending on the RBP. For example, we observed a significant increase in resolution in the splicing map for U2AF2, which resolved specifically to the intronic 3′ splice site region as opposed to overlapping the alternative exon (Fig. 7). However, for other RBPs (even those such as RBFOX2, which has previously been shown to crosslink directly to its in vitro binding motif [Weyn-Vanhentenryck et al. 2014]) we observed that using 5′ read ends yielded a similar structure with dramatically increased noise relative to using whole-reads (Fig. 7). Thus, these results suggest that this method can improve resolution for some RBPs (particularly those with highly specific splice site-proximal binding), but that factors with broader crosslinking and binding patterns may suffer an unacceptable loss of signal.

## DISCUSSION

The ability to profile both RNA processing and RBP association transcriptome-wide in vivo has revolutionized our ability to study the mechanisms of RNA processing. Integration of in vivo RBP targets identified by methods such as CLIP and RBP-responsive targets by knockdown or over-expression followed by RNA-seq or microarray, coupled with bioinformatics analysis techniques, has enabled the mapping of position-dependent regulatory principles for RBPs. For alternative splicing, this is typically referred to as a “RNA splicing map,” which visualizes the average binding signal across RBP-responsive AS events to simply summarize the role of that RBP on splicing regulation. Although many tools have been described to implement this approach, we found that incorporation of paired input data sets generated as part of eCLIP profiling required additional optimization. Therefore, we developed the RBP-Maps software package to enable users to implement a variety of normalization techniques and optimizations we observed to improve analysis of the ENCODE data resource.

Although this work focuses on describing the use of RBP-Maps (and the associated options) with respect to mapping the position-specific effect of RBP association on splicing regulation, the same approaches can be directly applied to position-specific regulation of 3′ or 5′ end processing, RNA stability and translation, or any other aspect of RNA processing regulated by RBPs. For example, polyadenylation analysis implicated splicing regulator NOVA in regulation of alternative polyadenylation (Licatalosi et al. 2008) and recently yielded insight into how binding of TARDBP/TDP43 shows differential regulation of alternative polyadenylation based on whether binding is close to, or further downstream from, a potential polyadenylation site (Rot et al. 2017). As it becomes easier to directly assay translation rates, RNA half-lives, and other aspects of RNA processing transcriptome-wide under RBP-modifying conditions, such RNA processing maps are likely to yield further insights into the complex regulatory code of RBP association.

## MATERIALS AND METHODS

### Identification of significantly altered splicing events

Data sets used included 203 RBPs with both eCLIP and knockdown/RNA-seq performed in the same cell type and released by the ENCODE project at https://www.encodeproject.org (Supplemental Table S1; Van Nostrand et al. 2017a). AS events were identified from rMATS JunctionCountsOnly files obtained from the ENCODE DCC (see accession identifier ENCSR413YAF for listings of all rMATS output files). Significant AS events were defined as having a *P*-value >0.05, FDR > 0.1 and change in exon inclusion level (also referred to as Percent Spliced In or |ΔΨ|) > 0.05. Elimination of overlapping splicing events was performed by identifying groups of overlapping AS events and selecting the event with the highest IJC among the overlapped events using the bedtools (v2.26) command merge (-o collapse -c 4) and pybedtools (v0.7.9). Positive IncLevelDifference (ΔΨ) indicates that the SE is more included upon RBP knockdown, while negative ΔΨ indicates that the exon is more excluded.

### Generation of control events

A number of background references for cassette exon comparisons were generated, including: “constitutive” cassette exons defined as exons in GENCODE v19 which had no exclusion observed in any of 29 scrambled shRNA control RNA-seq data sets in HepG2 or 29 in K562 (7351 events in HepG2 and 7888 in K562); “native” cassette exons defined as exons in GENCODE v19 with 0.05 < Ψ < 0.95 in at least half of control shRNA RNA-seq data sets for that cell type (1805 events in HepG2 and 2222 in K562); “included native” with inclusion > 0.67 in at least half of control data sets (1137 events in HepG2 and 1451 in K562); “central native” with 0.33 < inclusion < 0.67 in at least half of control data sets (256 events in HepG2 and 292 in K562); and “excluded native” with inclusion < 0.33 in at least half of control data sets (357 events in HepG2 and 439 in K562). All numbers reflect events remaining after removing overlapping events as described above.

### Splice map generation

Multiple approaches to generating RBP splicing maps were tested. For all methods, eCLIP signal (either read density or peak presence) was first identified for 350 nt windows flanking the relevant exon/intron boundaries, extending a maximum of 50 nt into each exon and 300 nt into each intron. For shorter exons (<100 nt) and introns (<600 nt), signal was only counted until the boundary of the neighboring feature. For cassette (skipped) exons, the relevant regions included the upstream, cassette, and downstream exon, creating four windows: the 3′ end of the upstream exon, the 5′ end of the cassette exon, the 3′ end of the cassette exon, and the 5′ end of the downstream exon, resulting in a total vectorized region of 1400 nt (350 × 4).

For peak-based splicing maps, each position within each vectorized region was marked as 1 if it was within a peak (requiring *P*-value ≤ 0.001 and fold-enrichment ≥ 8 in IP versus input), and 0 otherwise. These values are then summed and divided by the total number of events at each position to obtain the final splicing map. For Figure 2F, peaks with relaxed thresholds of fold-enrichment ≥ 2 were also used.

For read density-based methods, IP and input read density (normalized as RPM uniquely mapped, non-PCR duplicate reads) was identified at each position within the cassette exon region described above. For the background subtraction approach, input sample read density was subtracted from IP sample read density to result in difference values at every position through the event region. These values were then normalized in order to equally weigh each event by dividing the value at each position by the sum of absolute values across all 1400 positions (plus a pseudocount of one read, normalized to RPM, at each position) to obtain the normalized enrichment profiles for each event. For the relative information approach, per-position information was calculated using the equation

$$p_{i}\times \mathrm{lo}g_{2}\left(\frac{\;p_{i}}{q_{i}}\right),$$

where *p*_*i*_ and *q*_*i*_ are the per-position read probabilities at a given coordinate for IP and size matched input, respectively. To conservatively address positions with zero reads in either IP or input, a pseudocount of one read (normalized to total input read number) was added to each position before calculating IP and input read probabilities. Then, for both background subtraction and relative information approaches, values at each base across all events were sorted, removing the highest (2.5%) and lowest (2.5%) outlier values before calculating the mean across all events that is shown as the final splicing map.

To generate 5′ end splicing maps, density of 5′ read ends were identified using genomeCoverageBed (bedtools v2.26). Read end coverage was then used as input to the above pipeline, including background subtraction, outlier removal, and averaging across all events.

### Modeling significance between RBP-responsive and native events

Significance tests for peak-based maps were computed using the fisher_exact() function based on a 2 × 2 contingency table at each position *i* based on four conditions: RBP-responsive events with peak at position *i*, RBP-responsive events without peak at position *i*, native events with peak at position *i*, and native events without peak at position *i*.

Significance and confidence intervals for read density-based approaches were performed in two ways. For overall significance, a Kolmogorov–Smirnov test was performed comparing the outlier-removed normalized values for RBP-responsive events versus native events at each position using stats.ks_2samp() (Python scipy.stats module v1.1.0). To calculate significance and confidence intervals based on a bootstrapping approach, a random sample (with replacement) of *n* background events was selected, where *n* is the number of significant AS events in the test condition. These events then underwent outlier removal by filtering the top and bottom 2.5% of values, followed by calculating the mean at each position across all random events. This was repeated 1000 times to create a distribution of randomly sampled native event means at each position. By default, the 0.5th and 99.5th percentile values at each position were used to identify positions where RBP-responsive event maps were significantly different than native events. When multiple test conditions are present (e.g., included events and excluded events), this approach was performed separately for each, yielding a “max” and “min” value for each condition. For visualization of both knockdown-included and knockdown-excluded splicing maps on the same plot, the highest “max” and lowest “min” value was conservatively used to visualize error boundaries.

## SUPPLEMENTAL MATERIAL

Supplemental material is available for this article.

## Supplementary Material

Supplemental Material
