# Supplementary material for: RBP-Maps enables robust generation of splicing regulatory maps
Source: RNA. 2019 Feb;25(2):193–204. doi: 10.1261/rna.069237.118 (PMC6348990; doi:10.1261/rna.069237.118)
Supplement: Supplemental Material [file supp_25_2_193__index.html]

RBP-Maps enables robust generation of splicing regulatory maps — RBP-Maps enables robust generation of splicing regulatory maps — Supplemental Material 

# RBP-Maps enables robust generation of splicing regulatory maps

## Supplemental Material

- Supplemental\_Table\_S1.xlsx
